# The diagnostic value of (1,3)-β-D-glucan alone or combined with traditional inflammatory markers in neonatal invasive candidiasis

**DOI:** 10.1186/s12879-019-4364-x

**Published:** 2019-08-14

**Authors:** Junfei Guo, Yongbing Wu, Weiming Lai, Weiming Lu, Xiaoping Mu

**Affiliations:** Clinical Laboratory Department, Guangdong Women and Children Hospital, No. 521 Xingnan Road, Panyu, Guangzhou, 511400 China

**Keywords:** Invasive candidiasis, (1,3)-β-D-glucan, PCT, Hs-CRP, Neonate

## Abstract

**Background:**

Asymptom of invasive candidiasis (IC) and low positive rate of blood culture lead to delay diagnose of neonatal infection. Serum (1,3)-β-D-glucan (BDG) performs well in adult IC, but its use in neonatal IC is unclear. We evaluated the use of BDG, procalcitonin (PCT), high-sensitive C-reactive protein (hsCRP) or platelet count (PC) in neonatal IC.

**Methods:**

We collected the data of neonates admitted to our institute. Eighty neonates were enrolled, and divided into IC group, bacterial infection (BI) group and control (CTRL) group. We analyzed the difference of these indicators between groups, and generated Receiver operator characteristic (ROC) curve. The value of BDG in antifungal therapy efficacy assessment was also investigated.

**Results:**

The BDG level was higher in IC group compared with BI and CTRL group. *C. albicans* lead to significant increase of BDG compared with *C. parapsilosis*. IC group had highest hsCRP level and lowest PC. PCT level was similar between groups. ROC showed that BDG or hsCRP performs well in neonatal IC, the optimal cut-off for BDG was 13.69 mg/ml. Combined BDG with hsCRP, PCT and PC increased diagnostic value. Serum BDG level was decreased during antifungal treatment.

**Conclusion:**

Serum BDG performs well in identification of neonatal IC and in monitoring the antifungal therapy efficacy.

## Background

Neonates, especially the preterm one, are at high risk of invasive infection due to immaturity of immune system, high permeability of skin and mucosal, invasive medical care and prophylaxis antibiotic use. Fungus is one of the most causative pathogens of neonatal invasive infections. About 3% of preterm infants with birth body weight < 1500 g encounter IC [1]. Different fungus species could lead to invasive infection, but Yeast especially *Candida spp.* is the dominant isolated pathogens of invasive fungal infection. About 2.7% of early onset infections and 10.5% of late onset infections are invasive candida infection [2]. The mortality rate of IC is 28–41% [3]. Due to immaturity of immune system of the infants, the infected infants may have no signs or symptoms. Thus the diagnosis of IC is difficult. Isolate of fungus from normally sterile sites (blood, urine and cerebrospinal fluid) is the golden standard for diagnosis of IC. Due to small sample size collected and empirical antibiotic use, the sensitivity of culture is much lower in neonates than in adults. The positive rate of blood culture is < 50% in case of IC [4]. It’s urgent to develop new diagnostic methods for IC.

BDG is the component of the cell wall of many fungus species and the detection of serum BDG level could be used to diagnose the IC. Serum BDG level performs well in adult patients in intensive care units and onco-hematology department [5–10]. Though several studies investigating the diagnostic value of this marker in pediatric patients has been reported [11–14], its performance in neonates patients is still not well understood. Inflammatory markers such PCT, hsCRP have been widely used in diagnosis of bacterial infection, but their use in diagnosis of IC is under-researched.

Here, we present a retrospective study about IC in neonate patients at our institute. We investigated the value of BDG, PCT, hsCRP and PC alone or combined in diagnosing candidaemia, and the ROC curve of each indicator was generated to get the cut-off value. We further investigated the use of these indicators to distinguish candidaemia from bacteremia. We enrolled 30 neonatal cases of candidaemia in our study. To our knowledge, the studied population is relatively large compared with other related studies about the diagnostic value of BDG in neonatal population.

## Methods

### Study population and data collection

This is a retrospective study. The studied subjects were the neonates admitted to the NICU department of Guangdong Women and Children’s Hospital from January 2013 to January 2018, with available biological and clinical data. The demography data such as the birth body weight, gestational age (GA) and delivery mode were collected. The laboratory results of WBC and PLT count, PCT and hsCRP level, blood BDG level were collected from our laboratory system. The biological data of blood culture were also collected. The subjects were separated into three groups according to the results of blood culture: (a) the isolated pathogens were *candidiasis,* defined as invasive *candidiasis* group (IC); (b) the isolated pathogens were bacteria, defined as bacterial infection group (BI); (C) none pathogens were isolated, served as control group (CTRL). Those subjects without the data of BDG or blood culture data were excluded. The study protocol was approved by the Ethics Committee of Guangdong Women and Children’s Hospital. As this is a retrospective study and the patients scattered across Guangdong province (even southern of China), written consent to participate was difficult to obtain for some cases, but verbal consent to participate was collected from the parents/guardians of all the neonates via phone contact. The Ethics Committee of our institute approved the method of obtaining consent.

### Mycological examination

Blood cultures were conducted with BACT/ALERT® PF vials incubated in Bact/ALERT 3D system at 37°C for 5 days. Positive blood culture was transferred to sheep blood agar plate for isolation. The isolated pathogens were identified by mass spectrometry method with Bruker MALDI-TOF.

### Measurement of (1,3)-beta-D-glucan

The serum level of (1,3)-beta-D-glucan was detected with GKT assay kit (Gold Mountain River Tech Development Co., Ltd., Beijing,China) according to the manufacturer’s introduction. Briefly, 100ul serum sample was added to the dilution tube of the kit, the tube was incubated at 70°C for 10 mins followed by incubated at 10°C for 5mins; 200ul treated sample were transferred to reaction tube, and the reaction was read at 405 nm for 60mins. The concentration of BDG of each sample was calculated automatically using the calibration curve provided by manufacturer.

### Statistical analysis

Quantitative variables are presented as median and interquartile range (IQR: 25th percentile and 75th percentile) or mean and standard deviation. Categorical variables are described as percentages. The differences between groups were assessed by χ^2^ test for categorical variables and Mann-Whitney test for quantitative variables. ROC curve of BDG, CRP, PCT and PC alone or combined were generated with SPSS to determine the ability of these indicators, and cut-off values of these indicators were also calculated.

## Results

### Characteristics of the subjects

Eighty neonates (45 male and 35 female) were included into this study. The subjects were separated as IC (30 subjects), BI (25 subjects) and CTRL (25 subjects) group according to the result of blood culture. The obstetric history, clinical characteristics of the different groups are shown in Table 1. The gender distribution and delivery model were similar among different groups. Though the premature rupture of membranes rate was similar among different groups, the preterm labor rate was higher in IC and BI groups compared with CTRL group (Table 1). The median gestational age was 32 (IQR:30–36), 33 (IQR:28–37) and 36 (IQR:36–40) for IC, BI and CTRL group, respectively. The mean birth weight was 1840 ± 747 g, 1975 ± 654 g and 2215 ± 573 g for IC, BI and CTRL group, respectively.

**Table 1:** The characteristic of subjects

	Candidemia group (*n* = 30)	Bacteremia group (*n* = 25)	Control group (=25)
Obsteric Characteristics			
Premature rupture of membranes	5 (30)	4 (25)	2 (25)
Preterm labor	21 (30)	16 (25)	6 (25)
Vaginal delivery	22 (30)	17 (25)	19 (25)
Cesarean delivery	8 (30)	8 (25)	6 (25)
Neonatal			
Female (male)	11 (19)	13 (12)	11 (14)
Age^#^	16 h-28d (17d)	10 h-28d (15d)	15 h-28d (17d)
Gestational age (weeks)^#^	30–36 (32)	28–37 (33)	34–40 (36)
Birth weight (g)^*^	1840 ± 747	1975 ± 654	2215 ± 573

# indicates the data present as quartile and median

* indicates the data present as mean±std

### Pathogen distribution

In IC group, *C. albicans* was the most prominent isolated pathogen, followed by *C. parapsilosis*, and they accounted for 16 and 10 isolates, respectively. *C. glabrata* and *C.tropicalis* accounted for two isolates, respectively. The data of colonization of yeast in body sites such as digestive tract, respiratory tract or skin were not available. Coagulase negative staphylococcus (CNS) was the main isolates in BI group, followed by *Klebsiella pneumonia*.

### Inflammatory markers

**Fig. 1:**
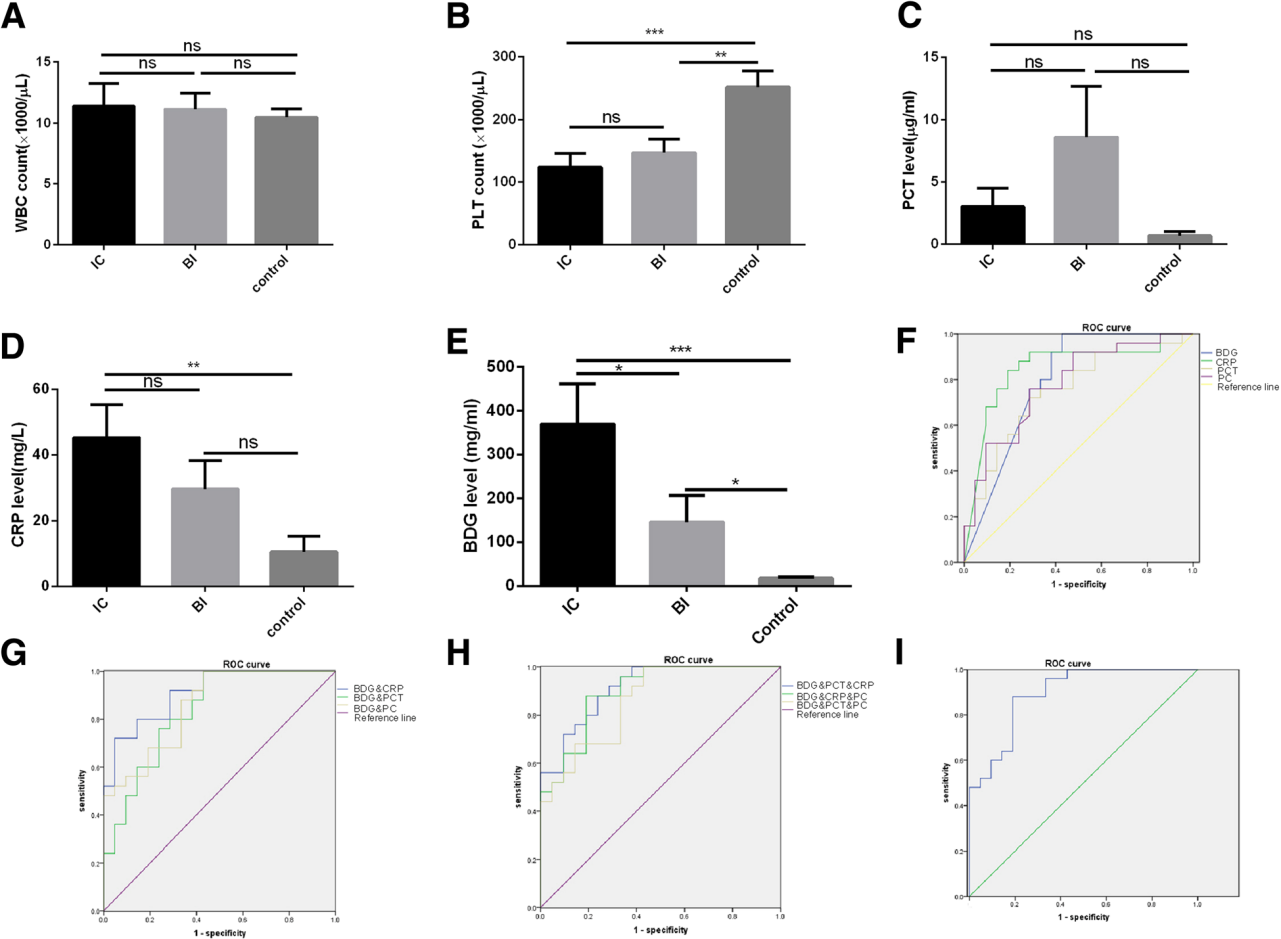
Comparison of the inflammation indicators between groups and their diagnostic value in IC. **a** The white blood cell count of the neonates in IC, BI and CTRL group. **b** The platelet count of the neonates in IC, BI and CTRL group. **c** The serum level of PCT in IC, BI and CTRL group. **d** The serum level of hsCRP in IC, BI and CTRL group. **e** The serum level of (1,3)-β-D-glucan was detected with GKT assay. **f** Showing is the ROC of BDG, CRP, PCT and PC alone in diagnose of IC. Blue line: BDG, green line: CRP, orange line: PCT, purple line: PC and yellow line: reference line. **g** showing is the combined ROC of BDG with CRP, PCT or PC. Blue line: BDG&CRP, green line: BDG&PCT, orange line: BDG&PC, purple line: reference line. **h** Showing is the combine ROC of three indicators. Blue line: BDG&CRP&PCT, green line: BDG&CRP&PC, orange line: BDG&PC&PCT, purple line: reference line. **i** showing is the combined ROC of BDG&CRP&PCT &PLT. ns: no significant, ***p < 0.001, **p < 0.01, *p < 0.05

The data of inflammatory markers such as hsCRP, PCT, WBC and PC were collected. None of the involved neonates encountered leukopenia (WBC < 1500/μl). Though the WBC count was increased in IC and BI groups compared with CTRL group, no statistical difference was found (Fig. 1a). The PC was significantly decreased in IC and BI groups compared with CTRL group (Fig. 1b). Eighteen of neonates in IC group encountered thrombocytopenia (PC < 150,000/ul), and 11 of them were severe thrombocytopenia (PC < 50,000/ul). Nine neonates in BI group encountered thrombocytopenia and four of them were severe thrombocytopenia. Only four neonates in CTRL group encountered thrombocytopenia, and two of them were severe thrombocytopenia. The PCT level of BI group was the highest followed by IC group, but there was no statistical differences between groups (Fig. 1c). The hsCRP level was significantly increased in IC groups compared with BI and CTRL group (Fig. 1d).

### The level of (1,3)-beta-D-glucan

The BDG level of IC group, BI group and CTRL group was 369.4 ± 91.88 mg/ml, 146.0 ± 60.80 mg/ml and 17.42 ± 2.932 mg/m, respectively. The difference between IC, BI and CTRL group was significant (Fig. 1e).

### The species-specific characteristic of candidaemia

We subdivided the IC group into *C. albicans*, *C. parapsilosis*, *C. glabrata* and *C. tropicalis* group according to the isolated pathogens, and compared the level of inflammatory markers and BDG between groups (Table 2). Compared with other candidas species, *C. albicans* related candidaemia had a higher level of BDG. The level BDG was 276.9 mg/ml (IQR:10–1000 mg/ml), 26.3 mg/ml (IQR:10–1000 mg/ml) and 21.7 mg/ml for *C. albicans*, *C. parapsilosis* and *C. glabrata*, respectively. The BDG level in *C. tropicalis* was unavailable. The serum level of hsCRP and PCT was no statistical differences between groups. Though no statistical difference, the PLT and WBC count was much lower in *C. parapsilosis* group compared with *C. albicans* group.

**Table 2:** The level of indicated factors in pathogen specific manner

Species	No.	No.with BBG	BDG (mg/ml)	CRP (pg/ml)	PCT (pg/ml)	PC (×1000/μl)	WBC (×1000/μl)
*C. albicans*	16	10	276.9 (10–1000)	24 (6.6–61)	0.51 (0.34–1.92)	129 (40–213)	10 (6.2–16)
*C. parapsilosis*	10	10	26.3 (10–1000)	32 (13.8–57)	0.51 (0.34–2.84)	101 (43–188)	8.2 (4.9–11.8)
*C. glabrata*	2	1	21.7	4.35	1.15	224	27.7
C.tropicalis	2	0	NA	69.2	0.35	220	6.2

### The value of BDG alone or combined with other inflammatory markers in diagnosing candidaemia

The ROC curve of BDG, hsCRP, PCT or PC alone was generated (Fig. 1f). The cut-off value of BDG was 13.69 mg/ml, with sensitivity of 76% [95% CI: 55–91], and specificity of 71.4% [95%CI: 49–89]. The positive likehood ratio of BDG was 2.66, and the area under the ROC (AUR) curve was 0.79 [95%CI: 0.65–0.93]. The cut-off value of hsCRP was 4.7 pg/ml, with sensitivity of 84% [95% CI: 64–95] and specificity of 81% [95%CI: 58–94]. The positive likehood ratio of hsCRP was 4.41, and AUR was 0.8 [95%CI: 0.71–0.97]. The cut-off value of PCT was 0.378 pg/ml, with sensitivity of 72% [95% CI: 51–88] and specificity of 71.4% [95% CI: 48–89]. The positive likehood ratio of PCT was 2.52 and AUR was 0.75 [95% CI: 0.61–0.90]. The cut-off value of PC was 169,000/μl, with sensitivity of 76% [95% CI: 55–91] and specificity of 71.4% [95% CI: 49–89]. The positive likehood ratio of PC was 2.66 and AUR was 0.78 [95%CI: 0.64–0.91]. Combined ROC of BDG with hsCRP, PCT or PC increased the diagnostic value (Fig. 1g). The AUR was 0.91[95% CI: 0.81–0.99], 0.83[95%CI: 0.71–0.95] and 0.85[95% CI, 0.75–0.96] for combined ROC of BDG with hsCRP, PCT or PC; Further combine of the markers did not significantly increase the diagnostic value (Fig. 1h and i).

### Diagnostic value of BDG alone or combined with other inflammation markers in distinguishing candidaemia from bacteraemia

**Fig. 2:**
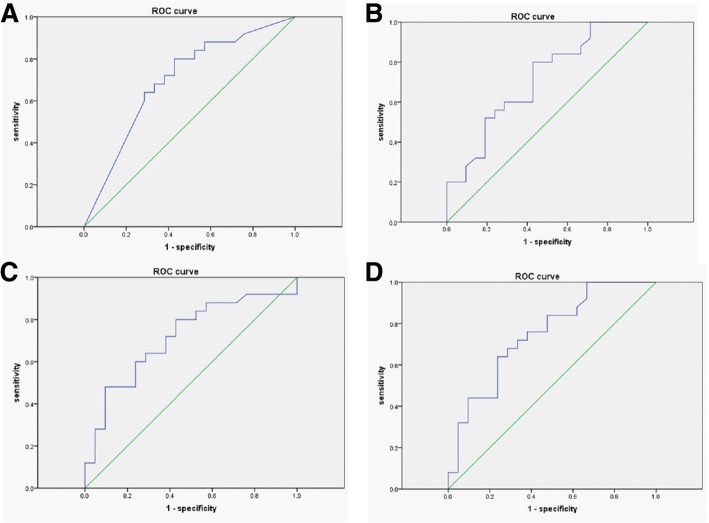
The ROC of single or combine indicators between IC and BI group. **a** Showing is the ROC of BDG in distinguishing candidaemia from bacteremia. **b**, **c** showing is the combined ROC of BDG with CRP, PCT, respectively, in distinguishing candidaemia from bacteremia. **d** Showing is the combined ROC of BDG, CRP and PCT in distinguishing candidaemia from bacteremia

We evaluated the diagnostic value of BDG in differentiating candidaemia and bacteremia. Serum BDG level at a cut-off value of 42 mg/ml could distinguish candidaemia from bacteremia, with sensitivity of 72% [95% CI: 51–88], specificity of 62% [95% CI: 39–82] and a positive likehood ratio of 1.89. The AUR was 0.70 [95% CI: 0.54–0.85] (Fig. 2a). Combined of hsCRP (Fig. 2b) or PCT (Fig. 2c) with BDG increased the AUR to 0.71 [95% CI: 0.56–0.86] or 0.715[95%CI: 0.56–0.86], respectively. Combined of CRP, PCT and BDG further increase the AUR to 0.748 [95%CI: 0.61–0.89] (Fig. 2d). Further combined of PC with CRP, PCT and BDG did not increase the AUR (data not showed).

### The use of BDG in evaluation of the effect of antifungal therapy

To assess the value of BDG in monitoring the effect of antifungal therapy, we collected series data of BDG of the same patient. The data of 11 patients was available. Antifungal therapy was began at 0* week when the blood culture was positive. As showed in Table 3, in general the serum of level of BDG was drop along with the antifungal therapy. We also found that the level of BDG increased at the beginning of antifungal therapy (patient 2, 5, 6, 8), this might reflect the transient release of BDG as the antifungal drug destroys the cell-wall of the Yeast. As some of the patients requested discharge before the infection was cured, the outcome of total patients was not available. None of the patient died during the hospitalization in our institute.

**Table 3:** Serial change of serum (1,3)-β-D-glucan along with anti-fungal treatment

Patient	Week	Culture result	BDG level (pg/ml)	Antifungal	Outcome
1	0^*^	C. albicans	1000	Fluconazole	survived
	1		954.6		
2	0^*^	C. parapsilosis	10	Fluconazole	survived
	1		78.17		
	2		70.95		
	3		71.27		
3	0^*^	C. albicans	789.5	Fluconazole	survived
	1		684.7		
	2		419.2		
	3		274		
4	0^*^	C. glabrata	21.7	Fluconazole	survived
	1		10		
5	0^*^	C. albicans	191	Fluconazole	survived
	1		292.5		
	2		126.3		
6	0^*^	C. albicans	726.3	Fluconazole	survived
	1		876.8		
	2		900.2		
	3		893.2		
	4		889.5		
	5		900.3		
	6		400.4		
7	0^*^	C. parapsilosis	1000	Fluconazole	survived
	1		10		
8	0^*^	C. albicans	10	Fluconazole	survived
	1		174.7		
	2		111.5		
9	0^*^	C. albicans	1000	Fluconazole	survived
	1		401.7		
	2		280.9		
	3		380.1		
	4		161.1		
	5		89.1		
10	0^*^	C. parapsilosis	1000	Fluconazole	survived
	1		378.2		
	2		100.2		
11	0^*^	C. parapsilosis	1000	Fluconazole	survived
	1		1000		
	2		1000		

* represents the week when the blood culture was positive

## Discussion

Although the general incidence of IC is low in neonatal population, the risk of IC is increased among preterm labor neonate [15, 16]. Asymptomatic of the infection and low sensitivity of blood culture often lead to the delayed diagnosis, which is associated with poor outcome [1, 17, 18]. Increase the volume of blood sample and repeated sampling could indeed increase the sensitivity of blood culture [18], but it’s difficult to conduct in neonatal population. Thus the development of non-culture based methods is of great important to identify patients at high risk of IC. (1–3)-β-d-glucan is the cell wall component of many fungus species, and serum level of BDG performs well in identify IC patients in adult population [6, 9, 10, 19, 20]. Our study indicated a great diagnostic value of serum BDG in identifying neonatal IC patients, consistent with several recent studies focused on neonatal and pediatric population [11–14]. Compared with traditional culture-base method, serum BDG has several advantages: small sample volume required; less time consumption (< 2 h); small impacts of prophylaxis antibiotic use.

In our study, the serum BDG levels were determined using the GKT assay (Gold Mountain River Tech Development Co., Ltd., Beijing, China), a novel assay to detect BDG in serum. The recommended cut-off value is > 60 pg/ml, but in our study we found that the optimal cut-off value of serum BDG was 13.69 mg/ml (Se:76%; Sp:71.4%) in our studied subjects. Our cut-off value is consistent with a recent study among children population < 14 years old using the GKT assay (including eight neonates) [13], the cut-off value of that study is 14 pg/ml. Our study and previous study argue that a new optimal cut-off value might be set for neonatal and pediatric population while using GKT assay, and more retrospective or prospective studies need to be conducted in neonatal population. Two other studies using Fungitell kit for serum BDG detection yet different cut-off value (86 pg/ml and 125 pg/ml) [11, 12]. As we known, these kits are developed from different horseshoe crab species; our results indicated that amebocytes from different horseshoe crab species may possess different affinities toward the BDG molecules. Thus it’s important to find out which kit is used while interpreting the result of serum BDG.

Serum BDG level was used for the diagnosis of IC in adult patients as recommended by the experts [6, 19]. Meta-analysis revealed a sensitivity of 57–97% and a specificity 56–93% among adult patients [5]. Searching PubMed with (1–3)-β-d-glucan and neonate targets few papers [11–14], indicating more investigation need to be performed in neonatal population. Low positive rate of culture, and short time consumption and small sample value of BDG test indicate BDG might be a good indicator to identify IC among neonatal population. Our study here shed some new insight into the use of serum BDG level in neonates.

Different fungus could lead to invasive infection among neonates. Previous study of Liu et al. showed that *C. parapsilosis* was the dominant isolated fungus [13]. In our study *C. albicans* was the main isolate fungus. Different composition of studied subjects and different location of the institute may be the reason for difference. The study of Liu et al. involved neonatal and pediatric patients and conducted in Shanghai, east of China. Our study involved only neonates and conducted in Guangzhou, south of China. Consistent with the result of Liu et al., we found that the patients infected with *C. albicans* have higher serum BDG level than the patient infected with *C. parapsilosis* (276.9 mg/ml (IQR:10–1000 mg/ml) vs 26.3 mg/ml (IQR:10–1000 mg/ml)). It seems that the cell wall of *C. parapsilosis* has smaller amounts of BDG, as suggested by their lower susceptibility to echinocandins (antifungals targeting BDG synthesis) [21, 22]. Several previous studies in adult population reported no difference of serum BDG level between *C. albicans* candidaemia and non- *C. albicans* candidaemia [22]. The reason for the difference between neonate/pediatric and adult is unknown right now.

In our studied subjects, no difference in WBC count between IC group and CTRL group was founded. More than half of IC neonates encountered thrombocytopenia and most of them were severe thrombocytopenia. Our result was consistent with that of Zhao et al. study [23], but different form the result of Marjorie et al. study [11]. These different might be due to the composition of the underlining diseases are different in these projects. CRP and PCT are two well documented indicators of bacterial infection. A few studies have been conducted to evaluate the use of these factors in diagnosis of IC in adult population [24–26]. In adult population, serum CRP level was increased while the serum level of PCT remained unchanged or slightly increased [24, 25]. Few studies focused on the use of CRP in neonatal IC patient, and no difference was found between IC neonates and control non-infected neonates [11, 12]. In our study, we found that the CRP level in IC group was significantly increased compared with CTRL group. The serum level of PCT in our IC group was slightly increased compared with CTRL group, consistent with the result of previous study in adult population [24]. Substantial increase of CRP and slightly increase of PCT is the main trends in our study, but more investigations are need before a conclusion could be made.

The treatment of fungemia is different from the treatment of bacteremia, thus differential diagnosis of these two kinds of infection is essential. Previous studies have reported that bacteremia could lead to false positivity of BDG detection [27, 28]. Here we found that compared with CTRL group, the serum BDG level was increased in bacteremia group, but decreased compared with IC group. Thus caution should be made while interpreting the result of BDG detection. The ROC of BDG indicated a cut-off value of 42 pg/ml (se:72%, sp.:62%) in differential diagnosis of candidaemia and bacteremia. Though no significant difference was found between groups, the PCT level in BI group was far higher than that of IC group. Sharp increase of PCT level is the characteristic of systematic bacterial infection [24]. How to quick distinguish candidaemia from bacteremia still need more investigation.

Several patients had multiple serum BDG level at different time points. The serum level of BDG in several patients was drop along with the treatment with antifungus drug, consistent with the result of previous study [11, 13]. We noticed, in several cases there is an increase of BDG level at the beginning of the treatment, the same phenomenon was observed in Liu et al. study [13]. Antifungals drugs destroy the cell wall of the fungus might be the reason of transient increase of BDG. Serum BDG level could use to monitor the effect of antifungals therapeutic.

## Conclusion

Serum BDG performs well in diagnosis of IC, and BDG combined with CRP, PCT or PLT count could further increase the diagnosis value. Significant increase of BDG and mild to severe increase of CRP and slightly increase or unchanged of PCT is characteristic neonatal IC. Neonatal IC may lead to severe thrombocytopenia. For neonatal population the optimal cut-off value of BDG was 13.69 mg/ml. Serum BDG level is a good indicator to monitor antifungals therapeutic effect.

## Data Availability

All the data of this project was presented in the manuscript. The raw data could be available via email if needed. The e-mail address is: gjunfei@sina.com.

## Abbreviations

AUR: Area under the ROC
BDG: (1,3)-β-D-glucan
BI: Bacterial infection
CNS: Coagulase negative staphylococcus
CTRL: Control
GA: Gestational age
hsCRP: High-sensitive C-reactive protein
IC: Invasive candidiasis
PC: Platelet count
PCT: Procalcitonin
ROC: Receiver operator characteristic curve
